# Increased Risk of Chronic Obstructive Pulmonary Disease in Patients with Systemic Lupus Erythematosus: A Population-Based Cohort Study

**DOI:** 10.1371/journal.pone.0091821

**Published:** 2014-03-12

**Authors:** Te-Chun Shen, Cheng-Li Lin, Chia-Hung Chen, Chih-Yen Tu, Te-Chun Hsia, Chuen-Ming Shih, Wu-Huei Hsu, Yen-Jung Chang

**Affiliations:** 1 Division of Pulmonary and Critical Care Medicine, Department of Internal Medicine, China Medical University Hospital and China Medical University, Taichung, Taiwan; 2 Division of Pulmonary and Critical Care Medicine, Department of Internal Medicine, Chu Shang Show Chwan Hospital, Nantou, Taiwan; 3 Department of Public Health, China Medical University, Taichung, Taiwan; 4 Management Office for Health Data, China Medical University Hospital, Taichung, Taiwan; 5 Department of Health Promotion and Health Education, National Taiwan Normal University, Taipei, Taiwan; Cardiff University, United Kingdom

## Abstract

**Background:**

There is increasing evidence that autoimmune disease is associated with development of chronic obstructive pulmonary disease (COPD). We aim to assess the relationship between systemic lupus erythematosus (SLE) and COPD risk in a nationwide population.

**Methods:**

We conducted a retrospective cohort study using the catastrophic illness registry of the Taiwan National Health Insurance Research Database (NHIRD). We identified 10,623 patients with SLE newly diagnosed between 2000 and 2010. Each patient was randomly frequency-matched with four people without SLE on age, sex, and index year from the general population. Both cohorts were followed up until the end of 2010 to measure the incidence of COPD. The risk of COPD was analyzed using Cox proportional hazards regression models including age, sex, index year and comorbidities.

**Results:**

The overall incidence rate of COPD was 1.73–fold higher in the SLE cohort than in the control cohort (17.4 vs. 10.1 per 10,000 person-years, 95% CI = 1.62–1.84). Age related analysis showed increased incidence of COPD with age in both SLE and control cohorts. However, adjusted HR maximum was observed in the youngest age group (adjusted HR: 4.33, 95% CI, 2.39–7.85) while adjusted HR minimum was witnessed in the oldest age group (adjusted HR: 1.19, 95% CI, 0.85–1.22).

**Conclusion:**

Patients with SLE have a significant risk of developing COPD than the control population. Based on the findings from this study, it can be hypothesized that in addition to cigarette smoke SLE may be a determining factor for COPD incidence. However, further investigation is needed to corroborate this hypothesis.

## Introduction

Systemic lupus erythematosus (SLE), an autoimmune disease that affects multiple organ systems, is more prevalent in women, particularly of child-bearing age. Although the exact mechanism of SLE initiation and progression is unknown, several risk factors, such as heredity, hormonal abnormalities, environmental pollutants, and viral infections, have been recognized as important contributors toward SLE development. An important clinical evidence of this disease is the presence of anti-double-stranded DNA (anti-ds DNA) autoantibodies, which are highly specific and can be used as diagnostic markers for disease activity and progression [1]. The annual incidence of SLE in adults ranges from 2.0 to 7.6 cases per 100,000 person-years in developed countries [2]. A recent population-based study from Taiwan found that the average incidence of SLE cases was 4.87 per 100,000 person-years between 2003 and 2008 [3].

Chronic obstructive pulmonary disease (COPD) is a slow progressive disease characterized by obstruction of airflow in the lungs due to chronic inflammation on the lining of airways. It is the 4^th^ leading cause of death worldwide, with reported prevalence rates between 5% and 13% [4], [5]. Although exposure to cigarette smoke plays a pivotal role in COPD development, a substantial group of patients with COPD has been identified as nonsmokers [6], [7]. Therefore, in addition to tobacco smoke, relevance of other contributing factors in COPD development cannot be ignored; thus, other contributing factors exist and one of them may be autoimmunity. The role of autoimmune pathology in the development and progression of COPD is becoming increasingly appreciated [8].

Smoking is a common environmental risk factor for COPD, but COPD development in patients with SLE may not be attributed to cigarette smoke alone. SLE may play an independent role in COPD incidence. Even smokers with SLE may display a different time course in COPD development compared with non-SLE individuals who were addicted to smoking. A recent epidemiological study from Israel has reported that rheumatoid arthritis, also an autoimmune disease, is significantly associated with COPD; the study was adjusted by controlling confounders including smoking [9]. Other study sources have revealed that the majority of patients with COPD contain increased levels of serum antibodies capable of interacting with self-antigens [10]–[16], and occurrence of antibodies to specific autoantigens correlates with disease severity [12]–[14], [16]. The mechanism of interactions between autoimmunity and COPD is still inconclusive.

The primary aim of our study is to determine whether a differential risk of developing COPD for adults exists with and without SLE by examining a relatively large population cohort in Taiwan. The results in this study were generated from a population-based retrospective cohort obtained from the National Health Insurance (NHI) system's database. To the best of our knowledge, this is the first nationwide population-based study evaluating the relationship between SLE and the risk of developing COPD.

## Methods

### Data Source

The National Health Insurance (NHI) program in Taiwan was first established in 1995, since then it has covered approximately 99% of Taiwan's population (23.74 million) [17]. The National Health Research Institute (NHRI) of Taiwan, in co-operation with the National Health Insurance Bureau (NHIB), has established a National Health Insurance Research Database (NHIRD), from which data were pooled for our study [18], [19]. The International Classification of Disease, 9th Revision, Clinical Modification (ICD-9-CM) was used for SLE diagnosis. Following regulations implemented by the Department of Health, the identity of each patient was encrypted for privacy and data security. This study was exempted from full ethical review by the International Review Board, China Medical University and Hospital Research Ethics Committee (IRB permit number: CMU-REC-101-012).

### Study Population

Patients certified with catastrophic illnesses including SLE were exempted from paying a copayment and therefore could be easily identified from the Registry of Catastrophic Illness Patient Database (RCIPD). A catastrophic illness is defined as a severe illness requiring advanced health care. Catastrophic illnesses usually involve high health care costs and may incapacitate the person from working, creating a financial hardship. All patients with SLE are categorized to have a catastrophic illness in the NHI system of Taiwan [3]. Patients certified with SLE catastrophic illness certification (ICD-9-CM code 710.0) as identified from the RCIPD covering a period of 10 years (2000–2010) were selected for this study. The index date for patients with SLE was assigned to be the date on which symptoms of SLE were first revealed. For comparison study, a non-SLE cohort control was randomly selected (4 for every patient in the SLE cohort) from the list of insured persons without a history of SLE and was matched for age, sex, and index year of SLE diagnosis. Patients in both cohorts with prior incidence of COPD (ICD-9 code 491, 492, and 496) or with missing information related to age and/or sex were excluded from this study.

### Outcome and Comorbidities

All study patients were followed until they were diagnosed with COPD as evident from the medical records. To measure the incidence of COPD, the SLE and non-SLE cohort were followed until COPD was diagnosed or death of the subject occurred or patients failed to follow-up or 31 December 2010, whichever came first. Comorbidities including hypertension (ICD-9-CM codes 401–405), diabetes (ICD-9-CM code 250), hyperlipidemia (ICD-9-CM code 272), coronary artery disease (CAD) (ICD-9-codes 410-414), cerebrovascular accident (CVA) (ICD-9-codes 430-438), and end-stage renal disease (ESRD) (ICD-9-CM code 585) were defined as diseases diagnosed before the index date.

### Statistical analysis

All statistical analyses were performed using SAS software, version 9.1.3 (SAS Institute, Cary, NC, USA). A two-sided statistical test with *p*<0.05 was considered statistically significant. The SLE cohort and non-SLE cohort data were compared using the Chi-square test for categorical variables and unpaired Student *t*-test for continuous variables. For comparisons between the SLE cohort and the non-SLE cohort, the incidence rate ratio (IRR) of COPD and 95% confidence interval (CI) were measured using the Poisson regression model. The univariable and multivariable Cox proportional hazard regression models were applied to measure the hazard ratio (HR) and 95% confidence interval (CI) of COPD incidence for the SLE cohort compared with that of the non-SLE cohort. Variables in the multivariable model included age, sex, hypertension, diabetes, hyperlipidemia, CAD, CVA, and ESRD. The Cox model was also used to estimate age, sex, and comorbidity specific HR. The Kaplan–Meier method was used to estimate cumulative incidence and the differences between the curves were tested with two-tailed log-rank test.

## Results

The demographics and medical comorbidities of patients enrolled in the study program are depicted in (Table 1). A total of 53,115 patients were enrolled in this study. The mean age (±SD) was 37.3±11.5 years for the SLE cohort and 37.1±11.9 years for the non-SLE control cohort.

**Table 1:** Demographic characteristics and comorbidities in patients with and without systemic lupus erythematosus.

Variable	SLE, n(%)		p value
	No	Yes	
	N = 42492	N = 10623	
Sex			
Female	37508(88.3)	9377(88.3)	0.99
Male	4984(11.7)	1246(11.7)	
Age, mean(SD)	37.1(11.9)	37.3(11.5)	0.07^#^
Age			
20–34 years	18596(43.8)	4649(43.8)	0.99
35–49 years	14012(33.0)	3503(33.0)	
50–64 years	6480(15.3)	1620(15.3)	
65+ years	3404(8.01)	851(8.01)	
Comorbidities			
Hypertension	2853(6.71)	2017(19.0)	<0.0001
Diabetes	1687(3.97)	540(5.08)	<0.0001
Hyperlipidemia	765(1.80)	646(6.08)	<0.0001
CAD	1023(2.41)	514(4.84)	<0.0001
CVA	1071(2.52)	635(5.98)	<0.0001
ESRD	197(0.46)	350(3.29)	<0.0001

Chi-square test, ^#^: Two sample t-test.

**Figure 1:**
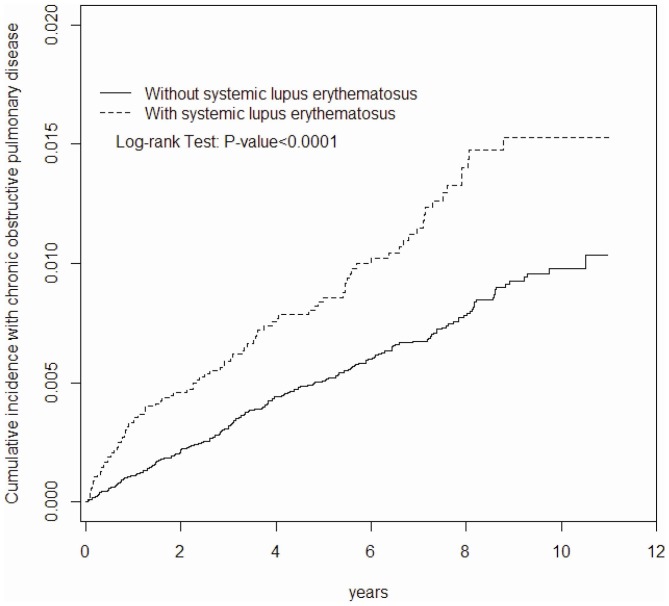
Cummulative incidence of COPD for subjects with and without systemic lupus erythematosus using the Kaplan–Meir method.

The majority of the subjects selected for this study were female (88.3%) and nearly (73.8%) of the subjects were aged 20–49 years (43.8%). When compared with the non-SLE control cohort, patients with SLE displayed higher proportion of comorbidities including hypertension (19.0% vs. 6.71%), diabetes (5.08% vs. 3.97%), hyperlipidemia (6.08% vs. 1.80%), CAD (4.84% vs. 2.41%), CVA (5.98% vs. 2.52%), and ESRD (3.29% vs. 0.46%). The mean follow up duration was 5.13±3.29 years for the SLE cohort and 5.32±3.24 years for the non-SLE cohort. The SLE cohort group reported higher rate of COPD incidence compared with the control cohort (17.4 vs. 10.1 per 10,000 person-years, 95% CI, 1.62–1.84, adjusted HR: 1.92, 95% CI, 1.50–2.44) (Table 2). The cumulative incidence of COPD by the end of follow-up period was approximately 0.55% higher in the SLE cohort than the non-SLE cohort (1.53% vs. 0.98%; *P*<0.001) (Figure 1). The sex specific risk analysis for both the cohorts revealed that both genders were equally susceptible in developing COPD (adjusted HR for women: 2.10, 95% CI, 1.55–2.83 vs. adjusted HR for men: 1.88, 95% CI, 1.24–2.86). Age related analysis showed increased incidence of COPD with age in both SLE and non-SLE cohorts. However, adjusted HR maximum was witnessed in the age range 20–49 years (adjusted HR: 4.33, 95% CI, 2.39–7.85) while adjusted HR minimum was observed in the oldest age group (>65 years) (Table 2). The multivariable Cox proportional hazard regression model showed an increased risk of COPD in patients with SLE with one of following characteristics: men (adjusted HR: 2.33, 95% CI, 1.84–2.94), older age (adjusted HR: 61.9, 95% CI, 34.7–110.7), diabetes (adjusted HR: 1.52, 95% CI, 1.16–1.99), CAD (adjusted HR: 1.86, 95% CI, 1.41–2.45), and CVA (adjusted HR: 1.99, 95% CI, 1.51–1.36) (Table 3).

**Table 2:** Sex- and age-specific incidence rates of COPD in subjects with and without systemic lupus erythematosus (SLE) and Cox model estimated hazard ratios for patients with SLE.

	SLE							
	No			Yes				
Variables (smoking rate %)‡	Event	PY	Rate^#^	Event	PY	Rate^#^	IRR*(95% CI)	Adjusted HR† (95% CI)
All	228	226007	10.1	95	54533	17.4	1.73(1.62, 1.84)***	1.92(1.50, 2.44)***
20–49 (25.0%)	21	176590	1.19	30	43844	6.84	5.75(5.34, 6.20)***	4.33(2.39, 7.85)***
50–64 (14.9%)	33	34499	9.57	21	7763	27.1	2.83(2.43, 3.29)***	2.38(1.37, 4.13)***
65+ (11.0%)	174	14918	116.6	44	2926	150.4	1.29(1.05, 1.58)*	1.19(0.85, 1.66)
Women	146	200870	7.27	63	48708	12.9	1.78(1.66, 1.91)***	2.10(1.55, 2.83)***
20–49 (5.6%)	17	159543	1.07	20	39726	5.03	4.72(4.37, 5.11)***	3.30(1.66, 6.58)***
50–64 (2.2%)	21	29555	7.11	13	6698	19.4	2.73(2.30, 3.24)***	2.20(1.09, 4.45)*
65+ (1.0%)	108	11773	91.7	30	2283	131.4	1.43(1.14, 1.81)**	1.45(0.96, 2.17)
Men	82	25137	32.6	32	5825	54.9	1.68(1.42, 2.00)***	1.88(1.24, 2.86)**
20–49 (42.8%)	4	17047	2.35	10	4117	24.3	10.4(8.17, 13.1)***	9.45(2.81, 31.9)***
50–64 (30.1%)	12	4944	24.3	8	1065	75.1	3.09(2.19, 4.38)***	2.81(1.14, 6.95)*
65+ (19.4%)	66	3145	209.9	14	643	217.7	1.04(0.68, 1.58)	1.01(0.56, 1.82)

Rate^#^, incidence rate per 10,000 person-years.

IRR^*^, incidence rate ratio.

† Model was adjusted for age, sex, and comorbidities.

‡ Current smoking rate of general population in Taiwan (%).

* p<0.05, ** p<0.01, *** p<0.001.

**Table 3:** Cox model with hazard ratios and 95% confidence intervals of COPD associated with systemic lupus erythematosus and covariates.

	Crude		Adjusted†	
Variable	HR	(95% CI)	HR	(95% CI)
Age				
20–34 years	1	(Reference)	1	(Reference)
35–49 years	3.74	(1.99, 7.03)***	3.62	(1.93, 6.79)***
50–64 years	12.1	(6.63, 22.2)***	9.03	(4.90, 16.6)***
65+ years	115.6	(66.0, 202.3)***	61.9	(34.7, 110.7)***
Sex				
Male	4.38	(3.48, 5.50)***	2.33	(1.84, 2.94)***
Female	1	(Reference)	1	(Reference)
Baseline comorbidities (yes vs no)				
SLE	1.72	(1.36, 2.19)***	1.82	(1.43, 2.31)***
Hypertension	9.53	(7.66, 11.8)***	1.28	(0.97, 1.68)
Diabetes	9.17	(7.20, 11.7)***	1.52	(1.16, 1.99)**
Hyperlipidemia	4.34	(3.06, 6.17)***	0.87	(0.60, 1.26)
CAD	13.1	(10.3, 16.7)***	1.86	(1.41, 2.45)***
CVA	12.2	(9.56, 15.6)***	1.99	(1.51, 2.620***
ESRD	2.20	(1.09, 4.43)*	0.67	(0.33, 1.36)

† Adjusted HR: multivariable analysis including for age, sex,

hypertension, diabetes, hyperlipidemia, CAD, CVA, and ESRD.

*p<0.05, ** p<0.01, *** p<0.001.

## Discussion

To the best of our knowledge, this is the first nationwide population-based study evaluating the relationship between SLE and COPD risk. In this study, there was a significantly higher incidence of COPD among patients with COPD than that in the general population. Further age related risk analysis indicated higher COPD incidence in aged patients although adjusted HR progressively decreased with age. This apparent contradictory finding could be explained by assuming aged patients may be exposed to cigarette smoke for a prolonged period of time or they could present with a large number of comorbid diseases. Therefore, the adjusted HR of oldest age group was not statistically relevant.

Another remarkable finding from this study revealed that the HR of developing COPD was comparatively higher (adjusted HR: 9.45, 95% CI, 2.81–31.9) in men aged 21–49 years with SLE than in men with similar age range in the control cohort. The differences in smoking rate might account for this observation. The smoking rate in men of the SLE cohort may be higher than that of the non-SLE cohort. Alternatively, SLE could be considered as an independent risk factor for COPD or smokers with SLE developed COPD within a short time frame. A recent public health report released from the Ministry of Health and Welfare of Taiwan, has reported that about 42.8% of males with age ranging from 20 to 50 years are smokers [20]. Although the NHI database does not contain information about personal smoking habits, we reasoned this value could be attributed to male smokers aged 20–50-years old in the non-SLE cohort. Therefore, to explain the occurrence of such a high HR, we hypothesized that either SLE directly causes COPD incidence or facilitates shortening of the disease course. However, further investigation is needed to corroborate this hypothesis.

For our study purpose, exposure to tobacco smoke is still considered as the most important confounding factor for COPD. Unfortunately, the NHI database does not provide any information on personal smoking habits. Freemer *et al* note that exposure to tobacco smoke has been associated with several autoimmune diseases [21]. Majka *et al* report the relationship between the development of SLE and cigarette smoking is less affirmative [22]. We have searched for previous publications which involved smoking rate among SLE patients and general population. We found that some studies with no significant difference between the two groups [23]–[26]. It is inconclusive that patients with SLE have a significant high smoking rate in all races. We roughly estimated the current smoking rate of non-SLE cohorts by considering data from public health reports obtained from the Ministry of Health and Welfare of Taiwan (Table 2). The overall smoking rate in the general population is 19.8% (males, 35.0%, females, 4.1%). Therefore, we considered combined effects of SLE and smoking for evaluation of increased incidence of COPD in this study.

Based on disease definition in the registry system as established by NHIRD of Taiwan, SLE is categorized as a “catastrophic illness” and patients diagnosed with SLE are entitled to receive the “catastrophic illness certification” issued by the government. The catastrophic illness certified patient is eligible for a great deal of discount benefits with regard to medical expenses. The certification process requires critical evaluation of medical records, serological, and/or pathological reports by experts specialized in the disease field [27]. The diagnosis of COPD is based on target history and requires comprehensive pulmonary function assessment. The criteria set forth by the GOLD guideline are usually followed in concluding the diagnosis of COPD [28]. A spot check is also performed regularly. Therefore, NHIRD provides a reliable data source for SLE and COPD occurrences. Moreover, any pre-existing condition of COPD would be detected by rheumatologists during comprehensive evaluation of patient's medical history while confirming SLE diagnosis. Therefore, patients diagnosed with COPD afterwards could be considered as new cases.

The exact link between inflammation and autoimmune disorders is not known. However, smoking serves as an important bridging link between the two diseases. Smoking is a well-known risk factor associated with several autoimmune diseases. Chronic exposure to cigarette smoke may be detrimental to the regular functioning of the body at the intracellular level. For example, smoking induces production of intracellular antigens [29]–[31], augments B cell autoreactivity and stimulates the proliferation of peripheral T-lymphocytes, and actively promotes generation of endogenous oxidative free radicals [29], [32]–[33]. Harmful toxins present in cigarette smoke may interact with DNA and cause genetic mutations, which ultimately lead to altered gene expressions resulting in autoimmune diseases [9]. COPD is a representative example of inflammatory disease. Although the exact underlying mechanism causing COPD has not been established, several mechanisms have been implicated in the pathogenesis of smoking induced COPD. Sirtuin 1 (SIRT1) is a deacetylase anti-inflammatory protein, the important functions of which are mediated by several different transcription factors (e.g., NF-kappaB). Decreased levels of SIRT1 and NF-kappaB activity have been reported in smokers exhibiting COPD conditions [34]. Vascular endothelial growth factor (VEGF) signaling is a crucial event associated with lung structure maintenance. Bronchiolar expressions of VEGF and VEGF2 receptors are significantly lower in smokers with COPD [35]. The toll-like receptors in the lung epithelium plays a pivotal role in host defense mechanism. Tobacco smoke has been identified as a potential modulator of expression of toll-like receptor 4 (TLR4) in respiratory epithelium [36].

The strength of our study lies in the use of population-based data that are highly representative of the general population. However, our study has several limitations. First, the National Health Insurance Research Database (NHIRD) does not contain detailed information regarding smoking habits, body weight index (BMI), diet preference, drug use, and family history of systemic diseases, all of which may be associated risk factors for COPD development. Second, the evidence derived from a retrospective cohort study is statistically less relevant than information obtained from randomized trials because of potential biases related to adjustments made for confounding variables. Despite our meticulous study design, biases resulting from unknown confounding factors may have affected our results. Third, all data in the NHIRD are anonymous. Thus, relevant clinical variables, such as serum laboratory data, pulmonary function tests, imaging results, and pathology findings were unavailable for patients in our study.

## Conclusion

To the best of our knowledge, our study is the first nationwide population-based study evaluating the risk of developing COPD in patients with SLE. Patients with SLE have a significant risk of developing COPD than the control population. Based on the findings from this study, it can be hypothesized that in addition to cigarette smoke SLE may be a determining factor for COPD incidence. However, further investigation is needed to corroborate this hypothesis.
